# Early detection and risk stratification in autoimmune-related interstitial lung disease: a state-of-the-art review

**DOI:** 10.1186/s12931-026-03538-0

**Published:** 2026-02-06

**Authors:** Paolo Delvino, Carlo Alberto Scirè, Valentina Bondi, Giulia Puccetti, Carlo Trentanni, Giovanni Franco, Umberto Zanini, Fabrizio Luppi

**Affiliations:** 1 School of Medicine and Surgery, University of Milano-Bicocca, Milan, Italy; 2 Rheumatology Unit, Fondazione IRCCS San Gerardo dei Tintori, Monza, Italy; 3 Epidemiology Research Unit, Italian Society of Rheumatology, Milan, Italy; 4 Respiratory Unit, Fondazione IRCCS San Gerardo dei Tintori, Monza, Italy

**Keywords:** Autoimmune rheumatic diseases, Interstitial lung disease, Rheumatoid arthritis, Connective tissue diseases, Vasculitis, Early detection, Risk stratification, Precision medicine

## Abstract

Interstitial lung disease (ILD) is a major pulmonary complication of autoimmune rheumatic diseases (ARD) and a leading contributor to long-term morbidity and mortality. Although ARDs share underlying immune dysregulation, the onset, radiologic phenotype, and clinical course of ILD vary substantially across individual diseases. Consequently, early detection and structured risk stratification at baseline and during follow-up have become essential elements of care for patients at risk of ARD-related ILD.

This review examines the principles and emerging strategies for early identification of ARD-related ILD, emphasizing the role of systematic clinical assessment, high-resolution computed tomography, and longitudinal pulmonary function evaluation in detecting early lung involvement. We discuss how radiologic patterns, functional measures, and serological profiles contribute to prognostic classification in different autoimmune contexts, with particular focus on the early identification of patients at risk of rapid ILD progression. Disease-specific ILD patterns are reviewed across major autoimmune conditions—including systemic sclerosis, idiopathic inflammatory myopathies, primary Sjögren’s syndrome, mixed connective tissue disease, systemic lupus erythematosus, rheumatoid arthritis, and anti-neutrophil cytoplasmic antibody-associated vasculitis—each characterized by distinct risk factors, distinct imaging findings, and divergent prognostic trajectories. Finally, we highlight emerging approaches to risk stratification, including integrated models that combine clinical, radiologic, and serological domains to mitigate the risk of disease progression and guide monitoring strategies.

Overall, current evidence supports a precision-medicine framework for ARD-related ILD, in which early recognition and individualized assessment of progression risk are crucial to improving outcomes and informing therapeutic decision-making.

## Background

Interstitial lung diseases (ILD) represent a major source of morbidity and mortality in patients affected by autoimmune conditions. Among autoimmune rheumatic diseases (ARD), several conditions are characterized by a clinically relevant risk of ILD. This group includes connective tissue diseases (CTD), such as systemic sclerosis (SSc), idiopathic inflammatory myopathies (IIM), primary Sjögren’s syndrome (pSS), mixed connective tissue disease (MCTD), and systemic lupus erythematosus (SLE), as well as rheumatoid arthritis (RA) and anti-neutrophil cytoplasmic antibody (ANCA)-associated vasculitis (AAV). Across this spectrum of ARDs, the prevalence, radiologic patterns, and clinical trajectories of ILD vary substantially [1].

Although SSc, IIM, and microscopic polyangiitis exhibit the highest burden of lung involvement, ILD can develop in any of the afore-mentioned ARDs, sometimes progressing silently and, not infrequently, constituting the inaugural manifestation of the disease [2, 3]. The pathogenesis of ARD-related ILD reflects a complex interplay between genetic susceptibility, environmental triggers, and dysregulated innate and adaptive immune responses. The underlying rheumatological condition and its specific autoantibody status are closely linked to distinct ILD phenotypes and provide valuable information for early risk stratification and prognostic assessment [4]. Given the potential for irreversible lung injury and the marked variability in disease course, early detection and structured risk stratification have become central to clinical management of ILD in ARDs. Current recommendations from the European Respiratory Society (ERS) and European Alliance of Associations for Rheumatology (EULAR) emphasize systematic screening and multidisciplinary evaluation, integrating clinical assessment, serological profiling, high-resolution computed tomography (HRCT) and longitudinal pulmonary function testing to refine diagnosis and guide therapeutic decisions [5]. The prognosis of ARD-related ILD is influenced by radiologic pattern, disease extent, underlying rheumatic disease activity, and coexisting comorbidities. Therefore, close collaboration between rheumatologists and pulmonologists is essential to ensure individualized care and timely intervention [6]. In this review, we synthesize disease-specific evidence across major ARDs, focusing on early detection strategies, characteristic ILD phenotypes, key serological and clinical predictors of progression, and evolving approaches to risk stratification aimed at optimizing long-term outcomes.

### Sources and selection criteria

A comprehensive literature search was conducted in PubMed and Embase to identify relevant studies on ARDs and ILD. The search covered publications from inception to August 2025 and used combinations of controlled vocabulary (Mesh) and free-text terms related to ILD, early detection, risk stratification, radiologic patterns, and ILD progression, together with disease-specific terms encompassing SSc, IIM, pSS, MCTD, SLE, RA, and AAV. Only English-language publications were included. Evidence from randomized controlled trials, prospective and retrospective cohort studies, case series, and pivotal mechanistic studies was considered. Particular emphasis was placed on studies addressing epidemiology, early detection strategies, HRCT phenotyping, prediction of progression and outcomes in ARD-related ILD. Abstracts from major rheumatology and pulmonology conferences were evaluated when they provided clinically relevant or emerging data.

### Connective tissue diseases

#### Systemic sclerosis

**Fig. 1:**
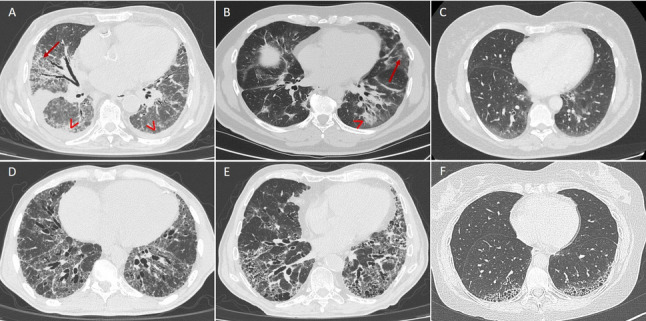
Main HRCT findings in autoimmune-related interstitial lung disease. *Panel ***A**: non-specific interstitial pneumonia (NSIP) pattern in a patient with systemic sclerosis, associated with early basal-predominant fibrosis (arrow) and bilateral pleural effusion (arrowhead). *Panel ***B**: combined NSIP and organizing pneumonia (OP), characterized by ground-glass opacities (arrow) and parenchymal consolidations (arrowhead) in a patient with anti-synthetase syndrome. *Panel ***C**: mild bibasal ground-glass abnormalities consistent with early NSIP in patient with primary Sjögren’s syndrome. *Panel ***D**: extensive fibrotic NSIP with prominent peripheral reticulations and fissural irregularities in a patient with mixed connective tissue disease. *Panel ***E**: usual interstitial pneumonia (UIP) pattern with predominant honey-combing and traction bronchiectasis in a patient with long-standing rheumatoid arthritis. *Panel ***F**: UIP pattern with moderate basal honey-combing abnormalities in a patient affected by microscopic polyangiitis

**Fig. 2:**
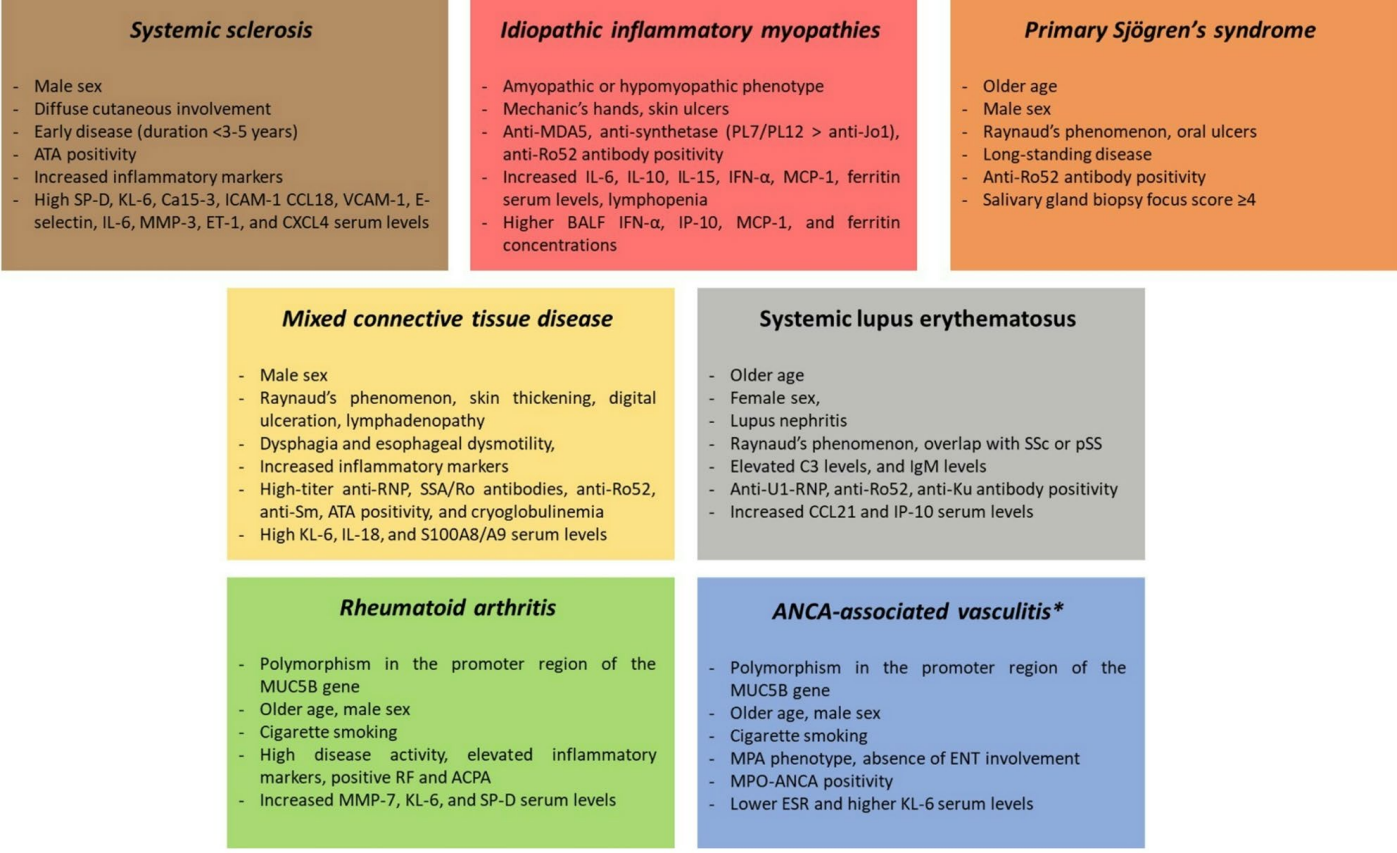
Disease-specific risk profiles in autoimmune-related interstitial lung disease

SSc is a complex autoimmune CTD characterized by vasculopathy, immune dysregulation, and progressive fibrosis of the skin and internal organs. Early ILD detection and structured risk stratification at baseline are central to modern management, as irreversible organ damage frequently develops before the disease becomes clinically overt [7, 8]. ILD is the leading cause of mortality in SSc, surpassing scleroderma renal crisis and cardiac involvement. Depending on the population and diagnostic approach, ILD occurs in 25–50% of patients, with the highest risk observed in diffuse cutaneous SSc and in individuals positive for anti-topoisomerase I antibodies (ATA) [9–11]. Although the clinical course of SSc-ILD is heterogeneous, progression of pulmonary disease contributes substantially to respiratory failure and accounts for the majority of SSc-related deaths [9, 12]. Survival at 5 and 10 years is significantly reduced in patients with ILD, particularly when pulmonary hypertension coexists [13]. Major prognostic indicators include reduced diffusing capacity for carbon monoxide (DLCO), impaired forced vital capacity (FVC), male sex, and elevated C-reactive protein (CRP) [12, 13]. Because ILD most often develops early in the disease course, systematic screening and longitudinal monitoring are essential. HRCT is recommended as a baseline assessment for all patients with SSc, as it is markedly more sensitive than pulmonary function tests (PFTs) or clinical evaluation alone for detecting early ILD [12, 14]. Both the American College of Rheumatology (ACR) and the American College of Chest Physicians (ACCP) guidelines advocate baseline HRCT screening in at-risk individuals, given its ability to identify parenchymal abnormalities even in patients with normal PFTs [14]. HRCT findings—particularly the extent of fibrosis—correlate strongly with subsequent functional decline and mortality (Fig. 1, panel A) [12, 15]. Quantitative imaging tools (qCT), including composite scoring systems and deep-learning-derived radiomic algorithms, further refine risk stratification by objectively quantifying fibrotic burden and pattern. High reticular scores and a greater likelihood of a usual interstitial pneumonia (UIP) pattern have been consistently associated with accelerated ILD progression during follow-up and poorer outcomes [16–19]. Biomarkers provide additional diagnostic and prognostic value in SSc-ILD. Serum markers, such as surfactant protein D (SP-D), Krebs von den Lungen-6 (KL-6) protein, and composite panels incorporating SP-D, Ca15-3, and ICAM-1 correlate with the presence and severity of ILD independent of age, sex, or lung function [20–23]. Prognostic biomarkers—including CCL18, VCAM-1, E-selectin, IL-6, MMP-3, ET-1, and CXCL4—are associated with increased risk of ILD progression and mortality, offering predictive information beyond traditional clinical and radiologic features [10, 20, 22]. Notably, elevated CCL18 levels predict significant FVC decline and transition to extensive disease during follow-up [20]. Integration of these biomarkers with clinical and imaging data enables more refined early risk stratification. The incorporation of clinical phenotype, autoantibody status, and imaging profile is of utmost importance in the personalized management of SSc-related ILD. Patients with diffuse cutaneous involvement, ATA positivity, early disease duration (< 18 months), and male sex are at highest risk for rapid ILD progression and poorer outcomes (Fig. 2). High-risk groups benefit most from early, intensive screening of ILD and timely initiation of immunosuppressive or antifibrotic therapy [10, 24].

SP-D: surfactant protein D; KL-6: Krebs von den Lungen-6; ICAM-1: intercellular adhesion molecule 1; CCL18: CC motif chemokine ligand 18; VCAM-1: vascular cell adhesion protein 1; MMP-3: matrix metalloproteinase-3, ET-1: endothelin-1; CXCL4: chemokine CXC motif ligand 4; anti-MDA5: anti-melanoma differentiation-associated protein 5 antibodies; MCP-1: monocyte chemoattractant protein 1, ATA: anti-topoisomerase I antibodies; CCL21: chemokine CC motif ligand 21, IP-10: Interferon-gamma-induced protein 10; MMP-7: matrix metalloproteinase-7; MPA: microscopic polyangiitis; MPO: myeloperoxidase; ESR: erythrosedimentation rate. *In most cases, interstitial lung disease precedes the clinical onset of ANCA-associated vasculitis.

#### Idiopathic inflammatory myopathies

IIM are a heterogeneous group of CTDs characterized by immune-mediated skeletal muscle inflammation and variable extramuscular involvement, including skin, joints, and lungs. Major clinical subsets comprise dermatomyositis (DM), polymyositis (PM), anti-synthetase syndrome (ASyS), inclusion body myositis (IBM), immune-mediated necrotizing myopathy (IMNM) and overlap myositis, each defined by distinct autoantibody profiles, pathogenetic pathways, and histopathologic patterns. Among extramuscular manifestations, ILD represents the most frequent and clinically relevant complication, affecting up to 40–60% of patients and standing as a major determinant of morbidity and mortality [25, 26]. Importantly, ILD may precede, coincide with, or follow muscle involvement, and often outweighs myositis activity in determining prognosis. Radiologically, IIM-associated ILD displays a spectrum of patterns reflecting the underlying immunopathology. Nonspecific interstitial pneumonia (NSIP) and organizing pneumonia (OP) predominate in ASyS and DM (Fig. 1, panel B), whereas rapidly progressive ILD (RP-ILD) is strongly linked to anti-melanoma differentiation-associated protein 5 (MDA5)-related disease. Fibrosing NSIP pattern with gradual gas-exchange decline is common in ASyS, while the UIP pattern is less frequent but associated with poorer outcomes [27]. Given its heterogeneity, early ILD identification is essential. Systematic respiratory evaluation at baseline—including PFTs, HRCT, and myositis-specific and myositis-associated autoantibody testing—is recommended to detect early ILD at diagnosis and prevent irreversible fibrotic remodeling [21]. Within the IIM spectrum, amyopathic or hypomyopathic DM (CADM) carries a particularly high risk of ILD, highlighting the dissociation between muscle and lung involvement and reinforcing the need for routine ILD screening even in patients with minimal or absent muscle manifestations. Anti-Ro52 antibodies are independently associated with ILD across IIM subsets and are strongly linked to fibrotic lung involvement, confirming their role as a robust serological risk marker irrespective of anti-synthetase antibody status [27].

A growing body of evidence indicates that anti-MDA5 disease is characterized by a hyperinflammatory pulmonary phenotype prone to RP-ILD. In a multicenter Chinese cohort of 168 patients with anti-MDA5 disease, serum IL-6 and lymphocyte count were identified as independent predictors of 6-month mortality and RP-ILD. Histopathological analysis revealed increased IL-6 expression within alveolar and vascular compartments and marked CD8 + T-cell infiltration, supporting a pathogenetic model driven by cytokine storm and cytotoxic T-cell injury [28]. These findings position IL-6 and lymphocyte dynamics as easily measurable biomarkers for early ILD risk stratification, enabling timely escalation of immunosuppressive treatment. Complementary evidence from Kurasawa and colleagues highlights a distinct cytokine and chemokine profile in anti-MDA5 disease, including elevated IL-6, IL-10, IL-15, and IFN-α, and markedly increased bronchoalveolar lavage fluid (BALF) concentrations of IFN-α, IP-10, MCP-1, and ferritin. BALF/serum ratio greater than 1 for IL-6, IL-15, IP-10, MCP-1, and IFN-α points to local pulmonary cytokine production driven by type I interferon signaling and macrophage activation. Higher serum IL-6, IL-15, MCP-1, and ferritin levels correlated with greater radiologic severity, supporting their utility as early markers of ILD development and progression [29]. In patients with anti-MDA5 disease, the assessment of PFTs at diagnosis is crucial for risk stratification and early ILD detection. In a multicenter cohort of 265 patients with ILD secondary to anti-MDA5-disease, baseline FVC emerged as the strongest predictor of short-term mortality: 6-month mortality was 15% in patients with FVC ≥ 50%, 47% in those with FVC < 50%, and 97% in patients too impaired to perform PFTs due to severe respiratory function compromise [30]. Baseline FVC thus provides a simple and robust tool for early recognition of patients requiring urgent therapeutic escalation.

A recent cluster analysis further refined early risk stratification by identifying three distinct inflammatory phenotypes in anti-MDA5 disease. The highest-risk cluster displayed marked systemic inflammation (elevated inflammatory markers, ALT, AST, LDH) and the highest prevalence of anti-Ro52 positivity, suggesting that combining anti-MDA5 and anti-Ro seropositivity defines a particularly aggressive phenotype predisposed to early RP-ILD [31]. Consistent associations between anti-Ro52, hyperinflammatory signatures (CRP, ferritin), and ILD severity have been corroborated in additional cohorts [32, 33].

In ASyS, autoantibody specificity is a powerful determinant of clinical expression and prognosis. In a large multicenter cohort of 233 patients, anti-Jo-1 positivity was associated with a systemic phenotype (myositis, arthritis, ILD), while anti-PL7 and anti-PL12 defined a lung-dominant phenotype characterized by ILD in > 90% of cases and minimal muscle involvement. Poor outcomes were associated with anti-PL7/PL12-positivity, age > 50 years, severe dyspnoea at diagnosis, and isolated ILD (Fig. 2) [34]. These observations were confirmed in a longitudinal cohort study the Johns Hopkins Myositis Center, demonstrating a more prevalent and severe baseline ILD, as well as lower FVC/DLCO in anti-PL7/PL12-positive patients compared with anti-Jo-1, and identifying black race as an independent predictor of ILD severity [35]. Taken together, these findings support an antibody-guided risk stratification strategy, with anti-PL7/PL12 seropositivity indicating the need for early HRCT at diagnosis and close functional monitoring.

#### Primary Sjögren’s syndrome

pSS is a chronic CTD defined by lymphocytic infiltration of exocrine glands and a broad range of systemic manifestations. Pulmonary involvement is increasingly recognized as a clinically meaningful extraglandular feature. Estimating its true prevalence remains challenging due to evolving classification criteria, but clinically significant lung disease occurs in approximately 9–24% of patients [36–38], while up to 75% of asymptomatic individuals demonstrate abnormalities on PFT , BALF, or HRCT [39]. Considering the multiorgan involvement of pSS, diagnosing pulmonary manifestations based solely on respiratory symptoms is challenging, as patients with mild ILD may be asymptomatic in the early stages of the disease. Fatigue, exertional dyspnea, and cough may also result from non-pulmonary causes such as anemia, chest wall involvement, joint disease, or muscle weakness. Nevertheless, respiratory symptoms may also reflect ILD progression, airway disease, vascular disease, or pleural involvement [40]. Pulmonary manifestations of pSS span the entire respiratory tract, with small airways disease—particularly follicular bronchiolitis—representing one of the characteristic histopathologic features. Airway involvement often precedes or coexists with ILD and contributes to cough, exertional dyspnea, and impaired quality of life [36]. Physical examination is often unremarkable; however, when ILD develops, fine bibasilar end-inspiratory “velcro-like” crackles may represent one of the earliest indicators of evolving ILD and warrant prompt investigations [41–44]. Chest radiography is generally considered insensitive for detecting ILD in pSS patients [45]. PFTs offer valuable information for early detection and longitudinal monitoring of ILD. Restrictive abnormalities with reduced FVC and total lung capacity (TLC), and normal forced expiratory volume in one second (FEV1)/FVC ratio suggest ILD [46, 47]. Conversely, an obstructive pattern characterized by increased TLC and a reduced FEV1/FVC ratio may reflect airway disease [48, 49]. The DLCO may be reduced in both conditions, due to ILD, pulmonary arterial hypertension (PAH), or emphysema [50]. ILD and airway disease may coexist in pSS patients; in such cases, spirometry may appear normal [51], although a disproportionate reduction in DLCO can occur [52]. Due to these limitations, HRCT remains the most sensitive diagnostic tool for identifying pSS-related ILD [4]. NSIP is the predominant radiologic and histopathologic pattern, reported in 28–61% of cases (Fig. 1, panel C) [46], while lymphocytic interstitial pneumonia (LIP)—historically considered the hallmark of pSS—now represents a minority of cases due to refined diagnostic criteria [53]. Other patterns include OP and, less frequently, UIP. Although subclinical abnormalities are often detectable, they do not necessarily progress to clinically significant ILD. HRCT should therefore be used systematically only in high-risk groups or when there is clinical suspicion of pSS-associated ILD, based on symptoms, physical examination, chest radiography, or PFT abnormalities [54]. Risk stratification at baseline in pSS-related ILD remains less codified compared with SSc or IIM. A recent systematic review identified only a few studies addressing risk factors for ILD in pSS patients. Older age, male sex, long-standing disease, elevated inflammatory markers, and seropositivity for ANA, anti-SSA, anti-SSB, or anti-Ro52 were consistently associated with a higher likelihood of ILD (Fig. 2). Clinical features including Raynaud’s phenomenon, oral ulcers, and salivary gland biopsy focus score ≥ 4 further delineated a phenotype at increased pulmonary risk [55]. Taken together, early detection of pSS-related ILD should rely on targeted HRCT prompted by symptoms, abnormal PFTs, auscultatory findings, or the presence of high-risk clinical or serological features.

#### Mixed connective tissue disease

MCTD is defined as a systemic CTD characterized by overlapping clinical features of systemic lupus erythematosus (SLE), systemic sclerosis (SSc), and polymyositis/dermatomyositis, in the presence of high titers of anti-U1 ribonucleoprotein (U1-RNP) antibodies, that distinguish MCTD from other CTDs [56–59]. Classification of MCTD relies on both clinical and serological criteria, with the Sharp and Kasukawa criteria being the most widely used [60, 61]. The most common and earliest clinical manifestations include Raynaud’s phenomenon, polyarthritis, puffy hands, myositis, sclerodactyly, esophageal dysmotility, and ILD. ILD is among the most frequent organ complications of MCTD, occurring in approximately 50–66% of patients [2, 62, 63]. The predominant radiologic pattern is NSIP, typically presenting with exertional dyspnea and cough, while clubbing and right heart failure are uncommon. HRCT most often reveals ground-glass opacities, fine reticulations, septal thickening and lower lobe predominance—features closely resembling SSc-related ILD (Fig. 1, panel D) [63–66]. Although ILD in MCTD may initially be mild or subclinical, early functional decline is a strong predictor of progression, emphasizing the need for systematic pulmonary screening at diagnosis. Multiple clinical and functional features have been associated with the development and progression of MCTD-related ILD, including Raynaud’s phenomenon, dysphagia and esophageal dysmotility, skin thickening, digital ulceration, lymphadenopathy at disease onset, and impaired baseline PFTs [67–73]. Serological predictors include elevated CRP, high-titer anti-RNP, SSA/Ro antibodies, anti-Ro52, anti-Sm, ATA, and the presence of cryoglobulinemia [67, 68, 71, 72, 74]. Male sex and the absence of arthritis have also been linked to more aggressive pulmonary disease [75]. Without timely detection and intervention, ILD secondary to MCTD frequently progresses, with up to 25% of patients developing severe pulmonary fibrosis within four years. Several circulating biomarkers have emerged as useful adjuncts for early detection of ILD and risk stratification of disease progression. KL-6, IL-18, and S100A8/A9 levels are consistently higher in MCTD-related ILD than in MCTD without pulmonary involvement and demonstrate additive diagnostic value [76, 77]. KL-6 levels also correlate with pulmonary function decline, reflecting fibrotic burden [76–78]. Additional markers, such as SP-A, SP-D, and chemokines (CXCL9, CXCL10, CXCL11), have been associated with ILD severity and may predict treatment responsiveness across CTD-ILD (Fig. 2) [77–80]. While none of these biomarkers is specific to MCTD-ILD, the combination of KL-6, IL-18, and S100A8/A9 may enhance early detection and refine risk stratification [76, 77].

#### Systemic lupus erythematosus

SLE is a chronic multisystemic autoimmune disease characterized by widespread immune-mediated injury affecting multiple organ systems, such as the skin, joints, kidneys, hematologic system, and serosal surfaces [81, 82]. Serologically, SLE is characterized by positive ANA, with anti-dsDNA and anti-Sm representing the most specific autoantibody markers. Given its multisystem involvement and protean clinical presentation, SLE shares several manifestations with MCTD, such as arthritis, cytopenias, mucocutaneous lesions, and serositis, positioning it within the broader overlap spectrum where pulmonary disease can also arise. ILD is a recognized but relatively uncommon complication of SLE, with prevalence estimates ranging from 1.2% to 6%, depending on population characteristics and diagnostic methodology [74, 83–85]. Among ILD subtypes, NSIP is the most frequently reported pattern, although UIP and OP patterns are also described [86–90]. LIP is uncommon, and some patients display mixed or atypical HRCT features that do not fit standard ILD classifications [91, 92]. Several demographic and clinical variables have been associated with early development of ILD in SLE, including older age at the time of SLE onset, female sex, lupus nephritis, elevated complement C3 levels, increased IgM concentrations, and the presence of anti-U1-RNP antibodies [84, 93, 94]. Additional factors linked to increased risk of ILD include Raynaud’s phenomenon and clinical overlap with other CTDs, especially pSS or SSc [85, 89]. A number of circulating biomarkers have been evaluated for early detection and risk stratification of SLE-associated ILD. While some chemokines such as CCL21 and IP-10 demonstrate high sensitivity and specificity for the occurrence of ILD in SLE, KL-6 and SP-D correlate with ILD severity and pulmonary function decline. Other autoantibodies, including anti-Ro52 and anti-Ku, have been proposed as potential risk factors, although their prognostic utility remains less well established [77–79, 95, 96]. Overall, while ILD is less frequent in SLE than in other ARDs, systematic evaluation of clinical risk factors, imaging findings, and serologic markers is crucial for timely recognition and appropriate monitoring of patients at increased pulmonary risk (Fig. 2).

### Rheumatoid arthritis

RA is a chronic systemic ARD that, although predominantly affecting the joints, is also associated with a broad spectrum of extra-articular manifestations, including ILD, which represents a significant determinant of prognosis [97]. The prevalence of clinically apparent ILD in patients with RA is estimated between 5% and 10%, but the presence of subclinical abnormalities detected by HRCT may occur at a substantially higher percentage, with recent meta-analyses reporting pooled prevalence rates approaching 19% [98, 99]. RA-ILD contributes significantly to morbidity and mortality, with a poorer prognosis compared to patients without pulmonary involvement [100]. Importantly, ILD can occur throughout the course of RA, including before the onset of articular disease and shortly after diagnosis during early RA [101], underscoring the critical importance of early detection strategies.

The pathogenesis of RA-ILD involves a complex interplay between genetic predisposition, environmental exposures, and immune dysregulation. Cigarette smoking, the most established environmental risk factor, promotes protein citrullination and loss of immune tolerance [102]. The MUC5B promoter variant (rs35705950), a well-known genetic risk factor for idiopathic pulmonary fibrosis (IPF), has been identified as an important predictor of RA-ILD, suggesting shared pathogenic mechanisms including abnormal epithelial repair and profibrotic signaling [103]. Autoantibodies, particularly high-titer anti-cyclic citrullinated peptide antibodies (ACPA) and rheumatoid factor (RF), may directly contribute to lung injury through immune complex deposition and complement activation [102, 104]. The predominance of the UIP pattern in RA-ILD, which often presents with a clinical-radiological phenotype indistinguishable from IPF, underscores the role of dysregulated wound healing and progressive fibrosis [105, 106].

Early identification of patients at risk is crucial for appropriate management and targeted monitoring. Several risk factors have been consistently associated with the development and progression of RA-ILD. Recent systematic reviews and meta-analyses have confirmed that male sex, older age, cigarette smoking, high disease activity, and the presence of high-titer RF and ACPA represent the most robust predictors of RA-ILD [98, 107–109]. Additional factors include rheumatoid nodules, elevated inflammatory markers (ESR, CRP), longer disease duration, older age at RA onset, and specific HLA-DRB1 shared epitope alleles [98, 108, 110]. Beyond UIP, other radiologic patterns such as NSIP and OP are also described, each carrying distinct prognostic implications [105, 106].

Current guidelines from the ACR and the ACCP recommend targeted screening for ILD in patients with systemic ARDs who present with risk factors, even in the absence of respiratory symptoms [14] and in particular in RA. The screening approach is based on clinical assessment, PFTs, and the use of HRCT in high-risk subjects. HRCT remains the most sensitive tool for early diagnosis, able to identify parenchymal abnormalities even in patients with normal PFTs [111]. Physical examination findings, such as fine bibasilar end-inspiratory “velcro-like” crackles, may represent one of the earliest clinical indicators of evolving ILD and warrant prompt investigation.

To facilitate systematic risk stratification and guide screening decisions, several clinical prediction models and risk stratification algorithms have been developed and validated at the time of diagnosis. The ESPOIR study investigators derived and independently validated a risk score for subclinical RA-ILD that incorporated male sex, increasing age, RA disease activity (DAS28), and the MUC5B promoter variant. This score demonstrated robust discriminative capacity for identifying patients at high risk of subclinical ILD before the onset of respiratory symptoms [101]. Moreover, Koduri and colleagues proposed a simplified four-factor risk score based on readily available clinical variables: smoking history (past or present), older age, positive RF, and positive ACPA [112]. Using a scoring system ranging from 0 to 9 points with a cut-off of 5, this model achieved a sensitivity of 86% and specificity of 58% (area under the ROC curve = 0.76), providing a pragmatic tool for identifying high-risk patients who should be considered for baseline HRCT and close monitoring in routine clinical practice.

The ongoing multinational ANCHOR-RA study is further investigating the prevalence of RA-ILD in populations selected based on recognized epidemiological risk factors, with the aim of refining screening strategies in real-world clinical settings. Complementary approaches incorporating circulating biomarkers—such as MMP-7, KL-6, and SP-D—have shown promise in enhancing early detection and baseline risk stratification [113].

Longitudinal monitoring of patients identified as high-risk is essential to detect progression and guide therapeutic decision-making. Serial PFTs, particularly FVC and DLCO, provide objective measures of functional decline and are recommended at regular intervals in patients with established or suspected ILD [14]. Progressive decline in FVC or DLCO, even in the absence of any new symptoms, may indicate ILD progression and warrant intensification of monitoring or therapeutic intervention. The integration of clinical, functional, radiologic, and biomarker data enables a multidimensional approach to risk stratification at diagnosis, allowing clinicians to identify patients at highest risk for rapid ILD progression and tailor monitoring and treatment choices accordingly (Fig. 2).

### ANCA-associated vasculitis

AAV comprises granulomatosis with polyangiitis (GPA), microscopic polyangiitis (MPA) and eosinophilic granulomatosis with polyangiitis (EGPA) [114]. These small-vessel vasculitides are characterized by multisystemic inflammation and necrosis, with predominant involvement of kidneys, upper and lower airways, peripheral nerves, and skin. Pulmonary disease is common and heterogeneous, including diffuse alveolar hemorrhage (DAH), granulomatous nodules, airways involvement, and various ILD patterns. Although the association between AAV and ILD has been recognized for decades [115, 116], its clinical importance has emerged more clearly only in recent years, with ILD now considered a central determinant of outcome in several AAV subsets [117]. Clinically, two groups are distinguished: ILD occurring in the context of overt AAV, and isolated ANCA-positive ILD, without systemic vasculitic manifestations.

#### ANCA-associated vasculitis-related interstitial lung disease

ILD affects approximately 20–45% of patients with AAV, with strong variability across populations and diagnostic methodologies [118, 119]. MPA shows by far the strongest association with ILD, particularly in patients with myeloperoxidase (MPO)-ANCA positivity, whereas ILD is less common in GPA and exceptional in EGPA [120, 121]. Importantly, ILD frequently precedes the clinical onset of vasculitis: in most contemporary cohorts, more than 80% of AAV-ILD cases had pulmonary fibrosis diagnosed months to years before systemic vasculitis [122, 123]. Patients with MPA-related ILD tend to be older than patients without lung involvement (65 vs. 55 years old, respectively), mirroring the age distribution of IPF [122, 124]. This epidemiologic parallel has fueled interest in shared pathogenetic mechanisms between MPO-AAV and fibrotic lung disease.

HRCT most commonly reveals a UIP pattern, accounting for 40–65% of MPA-related ILD cases (Fig. 1, panel F) [125]. Typical features include basal-predominant reticulations, traction bronchiectasis, and honeycombing, often indistinguishable from IPF. Some reports described peculiar findings such as “oddly shaped cysts” or combined pulmonary fibrosis and emphysema, suggesting subtle morphologic distinctions in AAV-related UIP [125]. Fibrotic NSIP is the second most common pattern, while OP is less frequent. Data from the RemIT-JAV cohort confirmed that ILD, particularly the UIP pattern, clusters strongly with MPO-ANCA positivity, whereas it is rare in PR3-ANCA disease [126]. A recent systematic literature review and meta-analysis reported that older age, male sex, smoking history, MPO-ANCA positivity, and elevated KL-6 levels were associated with an increased risk of ILD (Fig. 2). Conversely, ENT involvement, moderate-to-high BVAS scores, fever, and higher hemoglobin and albumin appeared to correlate with a lower probability of ILD [127].

The mechanisms driving ILD in AAV remain incompletely understood. The long-held hypothesis that recurrent subclinical alveolar hemorrhage promotes fibrosis [128] is currently challenged by the observation that pulmonary fibrosis typically predates vasculitis. An alternative view suggests that chronic fibrotic injury may itself promote MPO-ANCA development through persistent neutrophil turnover and loss of tolerance within damaged tissue [129, 130]. More recent models emphasize oxidative stress and neutrophil extracellular traps (NETs)-mediated injury: MPO-ANCA-activated neutrophils generate reactive oxidants [131] and release NETs [132] able to induce endothelial damage, stimulating fibroblast activation, promoting myofibroblast differentiation, and amplifying ANCA production [131, 133]. This creates a self-reinforcing inflammatory-fibrotic loop. Immunopathologic studies reveal a Th2/M2-skewed environment with elevated CCL2, CXCL13, and IL-13, consistent with a fibrosing immune phenotype [134]. Genetic predisposition further supports overlap with IPF: the MUC5B promoter variant, strongly linked to IPF, is enriched in MPO-AAV with ILD [135]. Integrating these observations, one plausible model posits that in genetically predisposed individuals with pre-existing fibrotic ILD, particularly UIP pattern, local injury may promote MPO-ANCA generation. Once initiated, ANCA-driven inflammation exacerbates fibrosis and may eventually progress to overt MPA [136]. Conversely, more inflammatory ILD patterns, such as NSIP or OP, may reflect primary pulmonary manifestations of AAV itself and tend to respond more favorably to immunosuppression.

Although the implementation of immunosuppressive strategies has remarkably improved survival in AAV, the coexistence of ILD remains one of the strongest determinants of adverse outcome. A recent meta-analysis demonstrated an almost threefold increase in mortality compared with AAV patients without ILD, with the highest risk observed in patients with a UIP pattern [137]. In a French cohort of AAV-related ILD, 5-year overall survival was 66%, compared with 84% in matched AAV controls without ILD [123]. Radiologic data from a large Chinese MPO-AAV cohort delineated a clear hierarchy of risk, with mortality highest in patients with DAH, followed by those with a UIP pattern and then an NSIP pattern [138]. Additional clinical variables further improve early risk stratification: older age, elevated inflammatory markers, baseline FVC < 80%, acute ILD exacerbations, long-term oxygen therapy, and use of immunosuppressants for induction [123, 139]. Notably, AAV relapse rates do not appear to be increased in AAV-related ILD [123], indicating that excess mortality in this population derives primarily from pulmonary complications rather than systemic disease activity. Taken together, these findings may suggest a pragmatic dichotomy within AAV-related ILD: a UIP pattern, which appears largely refractory or even vulnerable to immunosuppression, and non-UIP patterns, which tend to be more responsive to immunosuppressants, closely mirroring behavior observed in CTD-ILD.

#### ANCA-positive interstitial lung disease

Although AAV has not been included in the classification criteria for interstitial pneumonia with autoimmune features (IPAF) [140] and ANCA testing is not part of the standard ATS/ERS/JRS/ALAT ILD autoantibody panel [141], recent international ANCA testing recommendations now advocate selective screening in ILD populations [142]. Across multiple ILD/IPF cohorts, 5–10% of patients are ANCA-positive at ILD diagnosis [143, 144], and an additional proportion seroconvert during follow-up [143]. MPO-ANCA is consistently more frequent than PR3-ANCA in this setting. Notably, only MPO-ANCA-positive ILD appears to be at risk of evolving into overt MPA, with progression rates of 25–33% over 3–5 years [125, 144]. Among ANCA-positive ILD patients, UIP pattern is the strongest radiologic predictor of progression to systemic vasculitis. Observational data suggest that early immunosuppression has the potential to mitigate the probability of transition to AAV, although this requires prospective validation [125]. While transplant-free survival does not appear inferior to that of ANCA-negative IPF in some cohorts [144], MPO-ANCA-positive ILD has a distinct clinical course marked by an increased risk of vasculitic evolution, warranting structured surveillance.

## Conclusions

ILD represents one of the most impactful pulmonary manifestations of ARDs, and its early identification is critical for modulating the long-term disease course. Since ARD-related ILD displays wide variability in onset, radiologic appearance, and progression, clinicians increasingly rely on structured evaluation pathways to detect lung involvement before irreversible damage occurs. Multidisciplinary approaches that incorporate targeted history-taking, systematic imaging, and longitudinal assessment of PFTs allow clinicians to identify disease at an earlier stage and anticipate patterns of deterioration. Predicting prognosis in ARD-related ILD requires consideration of multiple domains. Although imaging features, functional trajectories, and immune profiles provide valuable information, none of them alone can fully describe disease behavior. Models that integrate these elements appear better suited to stratifying risk, capturing heterogeneity across ARDs, and informing the intensity of surveillance. The growing body of evidence points toward a transition from a uniform to a precision-based approach in ARD-related ILD. This shift emphasizes the importance of recognizing early signs of lung involvement, tailoring monitoring to predicted risk, and selecting therapies based on disease evolution rather than diagnosis alone. Key priorities for future research include validating multidimensional prognostic tools, strengthening the role of biomarkers capable of identifying progression risk, and testing early therapeutic interventions aimed at modifying the natural history of the disease. Progress in these areas will be crucial to improving outcomes across the full spectrum of ARD-related ILD.

## Data Availability

No datasets were generated or analysed during the current study.
